# Response to Commentary on the Prognostic Value of the Naples Prognostic Score After PCI in Acute Coronary Syndrome

**DOI:** 10.1002/clc.70485

**Published:** 2026-10-09

**Authors:** Mustafa Bilal Ozbay, Yasin Özen, Bülent Özbay

**Affiliations:** ^1^ Department of Medicine Penn Medicine Princeton Medical Center Plainsboro New Jersey USA; ^2^ Department of Cardiology Konya Selcuk University Medical Faculty Hospital Konya Turkey; ^3^ Department of Pulmonary Medicine, Faculty of Medicine Muğla Sıtkı Kocman University Muğla Turkey

Dear Editor,

We sincerely thank Dr. Cao and Dr. Xiang for their thoughtful and constructive comments regarding our recently published meta‐analysis evaluating the prognostic value of the Naples Prognostic Score (NPS) in acute coronary syndrome (ACS) patients undergoing percutaneous coronary intervention (PCI) [1, 2]. We appreciate the opportunity to clarify several methodological considerations and to further contextualize our findings.

We fully acknowledge the well‐recognized statistical limitation associated with dichotomizing continuous variables. Indeed, serum albumin, total cholesterol, neutrophil‐to‐lymphocyte ratio (NLR), and lymphocyte‐to‐monocyte ratio (LMR) are inherently continuous parameters and may exhibit non‐linear associations with clinical outcomes [3, 4].

However, our meta‐analysis synthesized data from previously published observational studies, all of which applied the validated, predefined categorical NPS framework (0–2 vs. 3–4) [5, 6, 7, 8, 9, 10, 11]. As a result, our analysis was constrained by the reporting structure of the original studies. Individual patient data were not available to permit modeling of continuous relationships, spline‐based analyses, or interaction testing between score components.

Importantly, our goal was not to modify the NPS but to assess how well it predicts outcomes in its current form as used in clinical research. While modeling biomarkers continuously may enhance risk discrimination at an individual level, categorical risk scores remain highly pragmatic tools in routine clinical workflows, particularly in time‐sensitive settings such as ACS [12].

We agree that future prospective investigations using continuous modeling, penalized regression, or machine learning approaches may refine prognostic calibration. Nevertheless, such efforts represent methodological extensions beyond the scope of a study‐level meta‐analysis.

The authors appropriately highlight that the NPS combines markers of nutritional status and systemic inflammation, potentially encompassing heterogeneous biological phenotypes. We agree that distinct mechanistic pathways, such as malnutrition‐driven vulnerability versus hyperinflammatory states, may coexist under the same total score. However, composite risk indices are intentionally designed to integrate multidimensional biological signals to enhance global risk stratification rather than to function as mechanistic classifiers.

Our study did not aim to delineate inflammatory phenotypes but to determine whether the total NPS is associated with adverse outcomes. In this regard, the pooled data demonstrated consistent associations with all‐cause mortality and no‐reflow despite inter‐study variability. Thus, while phenotypic refinement is an important future direction, it does not invalidate the prognostic signal observed with the current validated NPS construct.

We agree that observational data do not establish causality and have consistently framed our conclusions in associative, not causal, terms. Attenuation of associations after adjustment for disease severity does not invalidate prognostic value; many established cardiovascular scores, such as GRACE and TIMI, incorporate severity markers that function as risk integrators rather than mechanistic drivers [12, 13]. Our findings suggest that NPS stratifies risk across clinically meaningful outcomes, but whether NPS‐guided strategies improve outcomes requires prospective validation, as noted in our Discussion and Limitations.

Regarding clinical thresholds, included studies consistently used low (0–2) and high [3, 4] NPS categories, and our analysis assessed discrimination between these established groups. Defining treatment thresholds requires prospective calibration and decision‐curve analyses beyond the scope of aggregate‐level meta‐analysis. We do not propose replacing established ACS risk scores; rather, NPS may serve as a complementary, laboratory‐based risk stratification tool pending prospective validation.

We appreciate Dr. Cao and Dr. Xiang for this constructive discussion. Future research should focus on continuous biomarker modeling, phenotype‐based subclassification, prospective validation cohorts, and interventional trials evaluating NPS‐guided strategies. Our study represents an initial step in consolidating prognostic evidence, while mechanistic and interventional implications require well‐designed prospective investigations.

## Data Availability

Data sharing is not applicable to this article as no data sets were generated or analyzed during the current study.
